# TSH-independent release of thyroid hormones through cold exposure in aging rats

**DOI:** 10.18632/oncotarget.19851

**Published:** 2017-08-03

**Authors:** Gi Cheol Park, Ji Min Kim, Hee-Young Park, Ji Min Han, Sung-Chan Shin, Jeon Yeob Jang, Dawoon Jung, In Joo Kim, Jin-Choon Lee, Byung-Joo Lee

**Affiliations:** ^1^ Department of Otorhinolaryngology – Head and Neck Surgery, Samsung Changwon Hospital, Sungkyunkwan University School of Medicine, Changwon, Korea; ^2^ Department of Otorhinolaryngology – Head and Neck Surgery, Pusan National University School of Medicine and Biomedical Research Institute, Pusan National University Hospital, Busan, Korea; ^3^ Department of Internal Medicine, Samsung Changwon Hospital, Sungkyunkwan University School of Medicine, Changwon, Korea; ^4^ Department of Otorhinolaryngology - Head and Neck Surgery, Ajou University School of Medicine, Suwon, Korea; ^5^ Department of Internal Medicine, Pusan National University School of Medicine and Biomedical Research Institute, Pusan National University Hospital, Busan, Korea; ^6^ Department of Otorhinolaryngology – Head and Neck Surgery, Pusan National University School of Medicine and Biomedical Research Institute, Pusan National University Yangsan Hospital, Yangsan, Gyeongnam, Korea

**Keywords:** aging, thyroid, cold exposure, rat, Gerotarget

## Abstract

Thyroid function decreases and cold exposure response becomes impaired with increasing age. We investigated the age-related changes in thyroid structure and function and cold-induced changes in the thyroid activity of aging rats. Thirty-two male Sprague–Dawley rats were randomly divided into four groups (8 rats per group): young (7 months) and old (22 months) groups exposed to room temperature and cold stress. The active follicle ratio and serum free T3, T4 and TSH, and TSH receptor (TSHR) concentrations in the thyroid tissues of the rats from each group were compared. At room temperature, old rats had significantly lower active follicle ratio and free T3 and T4 concentrations than young rats. Furthermore, old rats displayed higher TSH level than young. Exposure to cold temperature led to significantly increased active colloid ratio and free T3 and T4 concentrations among old rats, but no significant differences were found among young rats. Additionally, no significant changes in the TSH and TSHR levels were observed after cold exposure in both young and old rats. Old rats have lower thyroid function than young rats under normal temperature. Aging rats are more susceptible to cold stress than young rats, and cold-induced thyroid activation occurs independently of TSH.

We investigated the age-related changes in the thyroid structure and function and cold-induced changes in the thyroid activity of aging rats. Aging rats have structurally less active thyroid follicles and functionally lower thyroid hormone levels than young rats. Furthermore, old rats are more susceptible to cold stress than young rats, and cold-induced thyroid activation occurs independently of TSH.

## INTRODUCTION

Control of the body temperature based on the surrounding environment is essential for proper physical activity performance and survival. When the body is exposed to cold, cutaneous vasoconstriction occurs to reduce heat loss, and metabolic heat production increases to maintain the core body temperature, followed by shivering [1]. However, the thermoregulation function continually declines as the age increases. Therefore, elderly people are at higher risk of hypothermia than younger ones, and mild cold stress may even cause their core temperatures to drop rather than extreme temperature [2, 3]. Elderly individuals have lower basal metabolic rate [4] and skeletal muscle volume, which causes shivering thermogenesis, than their younger counterparts, which may be the reason for the impaired thermogenesis in this population [1]. As is well known, the thyroid plays a very important role in the thermogenesis of homeothermic species with sympathetic nervous systems (SNS) [5–7]. Thyroid hormones directly act on the skeletal muscle to produce heat and the hypothalamus to stimulate the sympathetic nerves that innervate the brown adipose tissue to produce metabolic heat [8]. However, similar to the other endocrine organs, the thyroid gland also deteriorates with age, and subclinical thyroid dysfunction frequently occurs in the elderly [9, 10]. Therefore, such a decrease in thyroid function in the elderly can be assumed to be related to the cause of decrease in heat production. However, no studies have been reported on the relationship between aging thyroid and impaired thermogenesis.

The authors hypothesized that age-related thyroid dysfunction may be associated with the poor ability of elderly individuals to control their body temperature compared with young people and changes in the thyroid function after cold exposure would be different between the elderly and young people. In this study, we examined the age-dependent changes in the structure and function of the thyroid gland and compared the changes in the thyroid activity between the old and young rats after cold exposure.

## RESULTS

### Morphologic and functional changes in the aging thyroid

**Figure 1:**
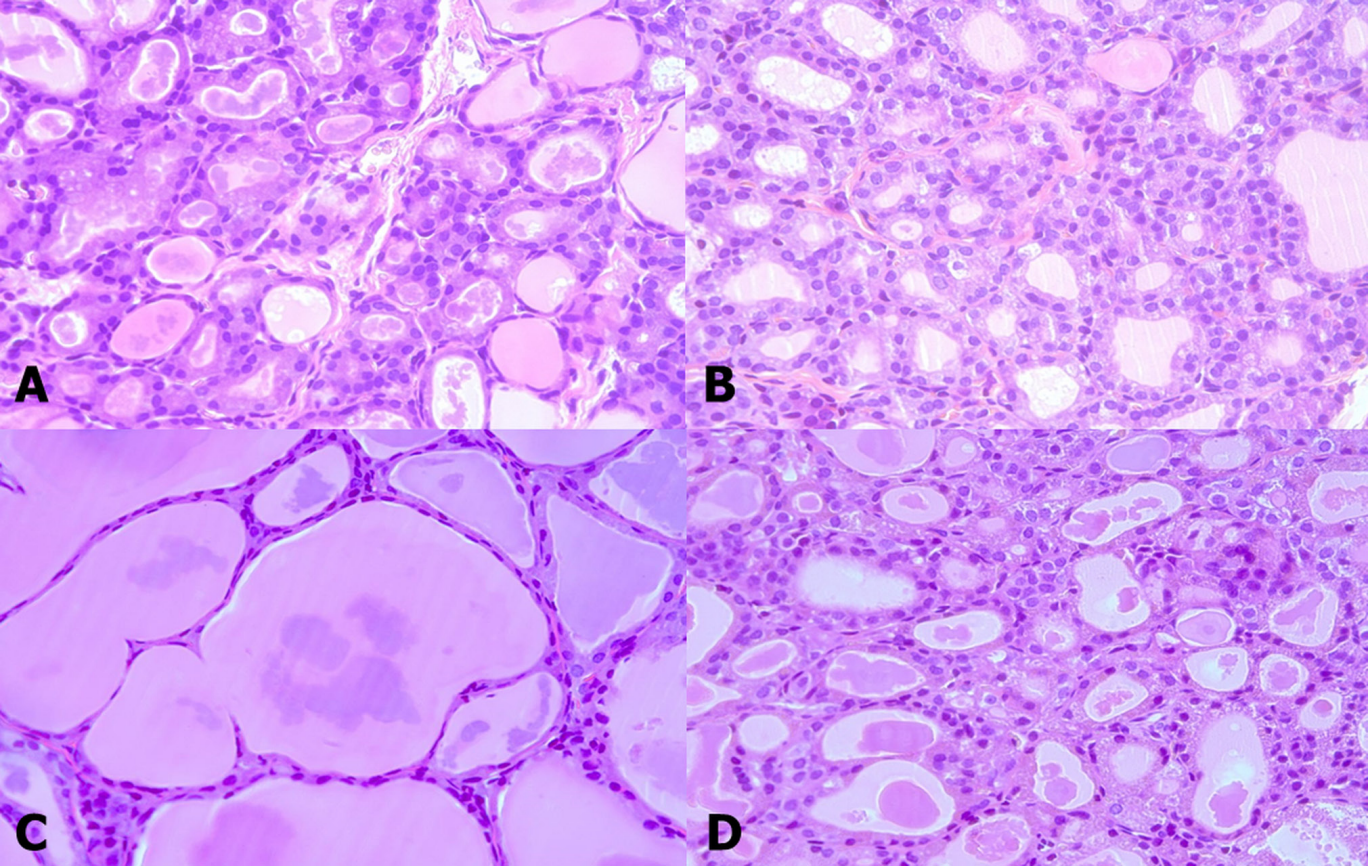
Thyroid follicle morphology of young (A, B) and old (C, D) rats exposed to room (A, C) and cold (B, D) temperatures At room temperature, old rats **C.** showed thyroid follicles with larger colloid amount but smaller and flatter cells than young rats **A.** The follicles of young rats did not significantly change **B.** However, the colloid amount was significantly reduced and the follicular cells were thickened again in old rats **D.**

**Figure 2:**
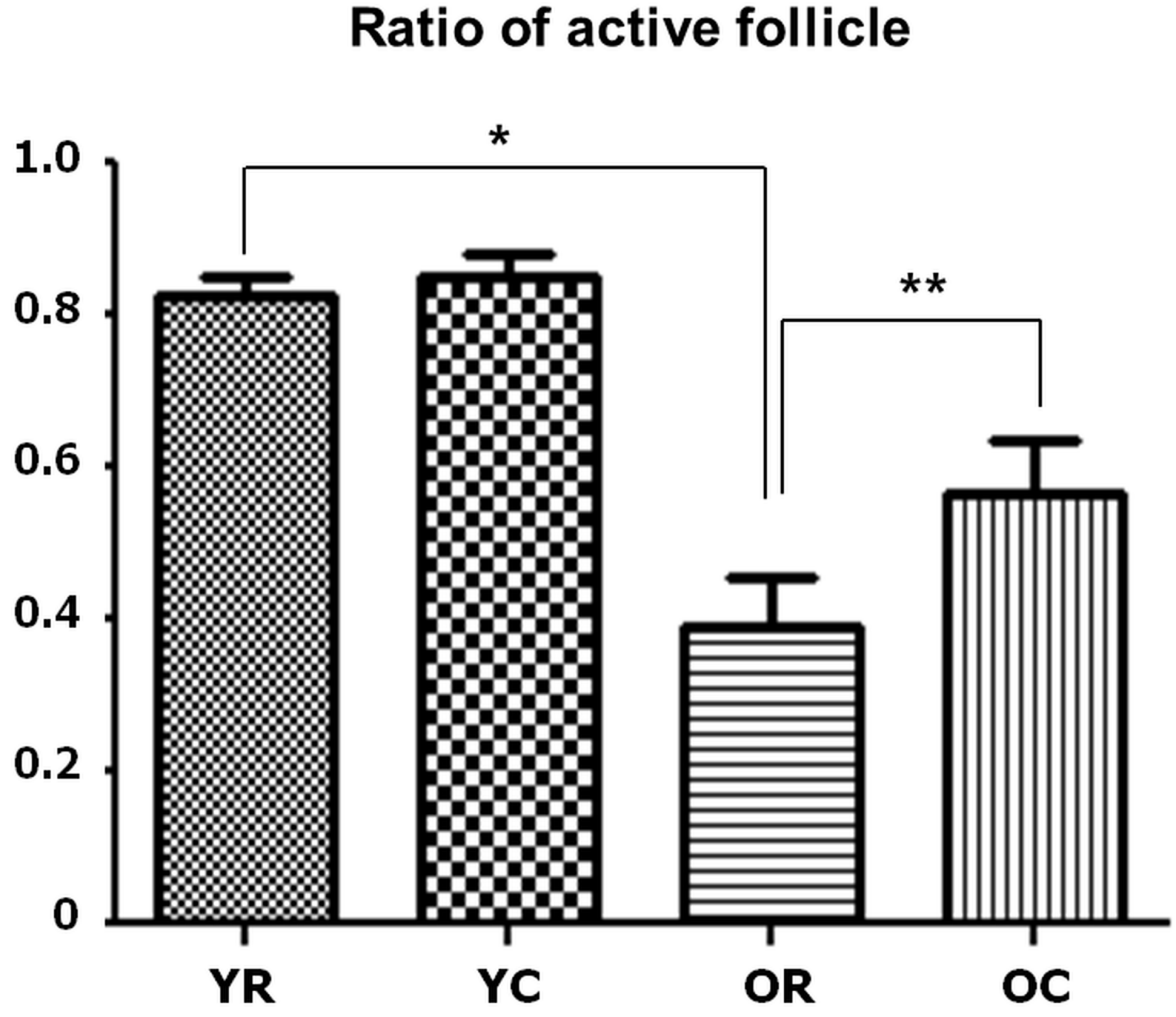
Changes in the active follicle ratios of young and old rats after cold exposure Old rats (0.375) had significantly lower active follicle ratio than young rats (0.825) at room temperature. After cold exposure, the active colloid ratio of old rats significantly increased from 0.375 to 0.5625, but no significant change in this ratio was observed in young rats. YC, young rats exposed to cold temperature; OR, old rats exposed to room temperature; YR, young rats exposed to room temperature; OC, old rats exposed to cold temperature. *p < 0.001, compared with YR and OR. **p = 0.002, compared with OR and OC. *Columns* and *error bars* represent mean ± standard deviation.

When we examined the structure of the basic thyroid follicle at room temperature, the follicular cells of young rats formed a thick cuboidal shape, and the colloids were not remarkably large (Figure 1A). However, the colloid size of old rats was significantly larger than that of young rats, and the follicular cells of the latter were small and flattened by the increased colloid amount (Figure 1C). Old rats (2.422 μm) had significantly smaller average follicular cell height than young rats (4.2307 μm, *p* < 0.001). Additionally, the colloid diameter of old rats (131.616 μm) is more than twice as large as that of young rats (61.166 μm, *p* < 0.001) (Table 1). The active colloid ratio of old rats was 0.375, which was significantly lower than that of young rats (0.825, *p* < 0.001) (Figure 2).

**Table 1:** Quantitative morphologic analysis of the thyroid follicles of male Sprague–Dawley rats after exposure to room and cold temperatures

	Young rats			Old rats		
Thyroid follicle	RT	CT	*p*^a^	RT	CT	*p*^a^
Cell height	4.2307 ± 0.71	4.203 ± 0.65	> 0.05	2.422 ± 1.06^b^	3.156 ± 3.14	0.047
Colloid diameter	61.166 ± 22.97	60.928 ± 12.97	> 0.05	131.616 ± 27.76^b^	119.952 ± 18.028	0.035

Abbreviations: RT, room temperature; CT, cold temperature

^a^One-way analysis of variance

^b^*p* < 0.001, compared with young rats exposed to RT

**Figure 3:**
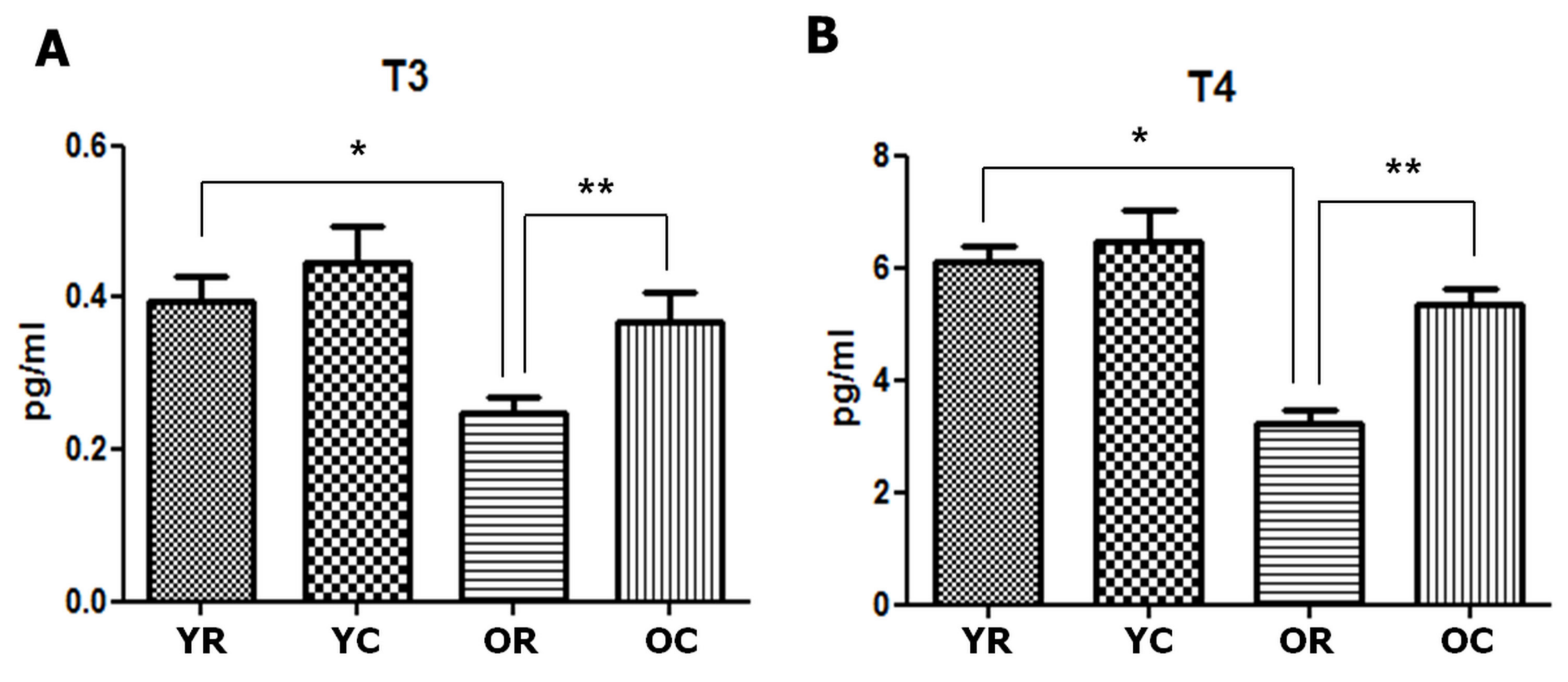
Changes in the thyroid hormones of young and old rats after cold exposure Free triiodothyronine (T3) **A.** and thyroxine (T4) **B.** concentrations were significantly lower in old rats (0.248 and 3.230 pg/mL, respectively) than in young rats (0.396 and 6.122 pg/mL, respectively). The free T3 and T4 concentrations of old rats significantly increased from 0.368 pg/mL to 5.330 pg/mL after cold exposure. YC, young rats exposed to cold temperature; OR, old rats exposed to room temperature; YR, young rats exposed to room temperature; OC, old rats exposed to cold temperature. *p = 0.001 (A) and *p* < 0.001 (B), compared with YR and OR. **p = 0.012 (A) and *p* < 0.001 (B), compared with OR and OC. *Columns* and *error bars* represent mean ± standard deviation.

Furthermore, old rats displayed lower thyroid hormone levels than young rats. The free triiodothyronine (T3) concentration of old rats was 0.248 pg/mL, which was significantly lower than that of young rats (0.396 pg/mL, *p* = 0.001). Moreover, old rats (3.230 pg/mL) had significantly lower free thyroxine (T4) concentration than young rats (6.122 pg/mL, *p* < 0.001) (Figure 3).

**Figure 4:**
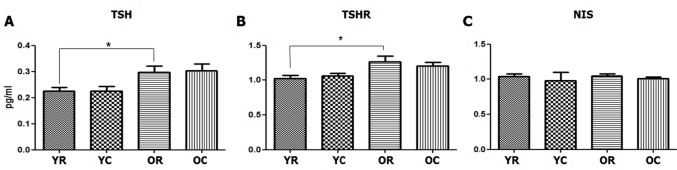
Changes in the serum TSH and TSHR and NIS levels of the thyroid tissues after cold exposure in young and old rats **A.** At room temperature, old rats had significantly higher serum TSH level (0.297 pg/mL) than young rats (0.224 pg/mL, *p* = 0.024). However, no changes in the serum TSH level was observed after cold exposure in both young and old rats. **B.** TSHR gene expression level of the thyroid tissues, which was determined through real-time polymerase chain reaction, was significantly higher in old rats than in young rats (*p* = 0.040). However, no change in the TSHR gene expression level was found after cold exposure in both young and old rats. Sodium-iodide symporter (NIS) **C.** showed no significant difference in all four groups (*p* > 0.05). YC, young rats exposed to cold temperature; OR, old rats exposed to room temperature; YR, young rats exposed to room temperature; OC, old rats exposed to cold temperature. *p = 0.024 (A) and *p* *=* 0.040 (B), compared with YR and OR. *Columns* and *error bars* represent mean ± standard deviation.

However, old rats (0.297 pg/mL) had significantly higher thyroid-stimulating hormone (TSH) level than young rats (0.224 pg/mL, *p* = 0.024). The thyroid-stimulating hormone receptor (TSHR) gene expression level of the thyroid tissues was also significantly higher in old rats than in young rats (*p* = 0.040). The finding on the increased TSHR gene expression was consistent with that on the increased TSH hormone level observed in old rats. Sodium-iodide symporter (NIS), which pumps iodine into the cell membrane in the cell membrane, showed no significant difference in all four groups (Figure 4).

### Cold-induced thyroid activation in aging rats

A significant change in the morphology of aging rats after cold exposure was observed. The thyroid follicle of young rats displayed minor changes in size and shape of the follicular cells or colloid even during cold exposure (Figure 1B). In old rats, the colloid size was significantly reduced from 131.616 μm to 119.952 μm (*p* = 0.035), and the follicular cells were thicker at room temperature than at cold temperature, with significantly increased height from 2.422 μm to 3.156 μm (*p* = 0.047) (Figure 1D) (Table 1). The active colloid ratio also significantly increased from 0.375 to 0.5625 in old rats (*p* = 0.002), but no significant change in this value was observed in young rats (Figure 2).

The thyroid hormone levels also showed similar pattern of change to the active colloid ratio. In old rats, the free T3 and T4 concentrations were 0.248 and 3.230 pg/mL, respectively. However, these values significantly increased to 0.368 (*p* = 0.012) and 5.330 pg/mL (*p* < 0.001), respectively, after cold exposure. In young rats, the free T3 and T4 concentrations slightly increased, although the change was not significant (*p* > 0.05). We also found that the active follicle ratio and free T3 and T4 concentrations all increased after cold exposure in old rats, although their values were still lower than those of young rats.

No significant changes in the TSH level after cold exposure was observed in both old and young rats (*p* > 0.05). Similarly, no significant change in the TSHR expression level of the thyroid tissues after cold exposure was found in both young and old rats (*p* > 0.05).

## DISCUSSION

This study have shown the functional and morphologic characteristics of the thyroid in aging rats and the changes in these characteristics after cold exposure. The ratio of the active thyroid follicles and level of thyroid hormones, such as free T3 and T4, decreased, whereas the concentrations of TSH and TSHR increased in aging rats compared with young rats. After cold exposure, the proportion of active follicles was increased in old rats, and their free T3 and T4 concentrations were also elevated. However, the TSH or TSHR level of these rats did not change.

The thyroid follicle is a functional unit consisting of colloid and follicular cells. Colloid is a material reservoir that is used in the production of thyroid hormones in the inner cavity of the follicle, which stores the thyroid hormones itself. The follicular cells surround the colloid, absorb iodine from the surrounding blood, and produce thyroid hormones. If the thyroid is resting, the colloid amount is relatively high, so the follicular cells are flattened. When the secretory function of the thyroid hormones become active, the colloid amount decreases, and the cells pressed down by the colloid become plump again [11]. Therefore, we can evaluate the thyroid gland activity by analyzing the shape of the thyroid follicle. Indeed, the thyroid activation index (TAI), which calculates the relative volume ratio of the colloid and follicular cells in the thyroid follicles, is a good indicator of the functional activity of the thyroid gland and has a positive correlation with the thyroid hormones [12, 13]. In this study, thyroid follicles of the aging rats had considerably larger colloids but smaller and flatter follicular cells than those of young rats. The ratio of the active follicles, which is used to quantitatively calculate the shape of these follicles, was also significantly lower in old rats than in young rats. Furthermore, the change in the free T3 and T4 concentrations also have the same pattern of change as that of the active follicle ratio. However, old rats have significantly higher TSH level than young rats. Understanding on the mechanism behind the low levels of thyroid hormones despite the high levels of TSH in elderly individuals is still lacking [14–16]. One hypothesis is that this phenomenon may be associated with age-related decrease in the sensitivity of the thyroid gland to TSH [14]. However, this hypothesis does not appear to be correct because the TSHR level detected in the thyroid tissues is higher in aging rats than in young ones in this study.

Old rats exposed to cold temperature displayed thyroid follicles with decreased colloid amount, thickened follicular cells, and significantly increased active follicle ratio compared with those expose to room temperature. The thyroid functions also significantly increased along with the change in the active colloid ratio after cold exposure. However, young rats did not show any significant morphologic or functional change despite exposure to cold, but they still had higher active follicle ratio and thyroid hormone levels than old rats. The differences in the thyroid function changes after cold exposure between young and old rats indicate that aging rats respond first to the cold rather than the young ones because the former have reduced ability to produce metabolic heat through lowering of the basal metabolic rate compared with the latter; moreover, although cold adaptation of the thyroid occurs first in aging rats [17–21], their heat production capacity is likely to be still insufficient compared with the young rats [22, 23].

During cold adaptation, the thyroid hormones synergistically interact with the sympathoadrenal system at various stages to induce thermogenesis [5, 24, 25]. The thyroid hormones modulate the SNS and causes heat production in the brown adipose tissues [26]. Meanwhile, the neurotransmitters from the SNS can also activate the thyroid [27, 28]. The thyroid follicles are innervated by various nerve fibers, including the sympathetic fibers that contain norepinephrine [29]. Norepinephrine stimulates the mitosis of the thyroid gland, and the thyroid hormones actually become elevated when the adrenergic activity is increased [30, 31]. These changes occur even in the absence of changes in TSH levels, which indicates that the sympathetic innervation of the thyroid exerts a direct action independently of the TSH [32]. In this study, both the TSH and TSHR levels remain unchanged, even when the free T3 and T4 concentrations increased after cold exposure in aging rats. Thyroid activation was not mediated through the TSH-thyroid axis but through other pathways innervating the thyroid. SNS activation due to cold stress may be assumed to be one of the factors causing thyroid activation.

We did not assess the changes in body temperature and brown adipose tissue, which play an important role in thermogenesis in this study. Therefore, we could only show an increase in thyroid activity after cold exposure and could not directly explain the relationship between thyroid activity and thermogenesis. Additionally, as previously mentioned, the mechanism through which the thyroid is activated by specific pathways remains to be elucidated, although cold-induced thyroid activation in aging rats has been found to be TSH-independent. Further research in this aspect will be needed in the future.

In conclusion, aging rats have structurally less active thyroid follicles and functionally lower thyroid hormone levels than young rats. Furthermore, old rats are more susceptible to cold stress than young rats, and cold-induced thyroid activation occurs independently of TSH.

## MATERIALS AND METHODS

### Animals

Thirty-two specific, pathogen-free male Sprague-Dawley rats (16 young [7 months] and 16 old [22 months] rats) were obtained from Samtako (Osan, Korea). The rats were divided into four groups (8 rats per group): young (*n* = 8, 7 months) and old (*n* = 8, 22 months) groups exposed to room temperature (21 °C) and those (*n* = 8, respectively) exposed to cold stress. Each group was weight matched at the beginning of the study so that the initial body weights were similar. To investigate the effects of acute cold stress on the aging process, young and old rats were exposed to a temperature of 4 °C for 5 h before they were sacrificed. The animal protocol used in this study was reviewed and approved beforehand by the Institutional Animal Care and Use Committee of Pusan National University with respect to ethicality and scientific care.

### Tissue preparation and morphologic analysis of the thyroid follicles

The whole thyroid glands were isolated and prepared for overnight fixation in 4% formalin. The thyroid glands were dissected into 4-5 mm wide strips, which were then subjected to tissue processing and embedded in paraffin. Cross-sections (8 μm thickness) were obtained at four predefined points and placed on glass slides. The sections were prepared for hematoxylin-eosin staining. For the staining analyses, the slides were deparaffinized with xylene and then hydrated through a series of washes with 100%, 85%, 75%, and 50% ethanol and finally water.

Morphologic analysis was performed on every third serial section of the tissue. The shape and height of the follicular epithelial cell and the internal diameter of the colloid were measured. Additionally, the morphologic changes in the thyroid gland were also quantified through stereological analysis [11]. The histometric TAI, which has been proposed as the ratio of the volume density of the follicular epithelium to that of the colloid, was also determined [12, 13]. The thyroid follicle was considered as an active follicle if its TAI was higher than 2. The ratio of the active follicle to the entire follicle was also calculated.

### TSHR and NIS in the thyroid tissues

The tissue RNA was extracted using the TRIzol Plus RNA Purification System (Life Technologies, Rockville, MD, USA). Reverse Transcription Kit (Applied Biosystems, Foster City, CA, USA) was used to perform reverse transcription in accordance with the manufacturer's recommended reaction protocol. Real-time polymerase chain reaction (PCR) was conducted using SYBR Green PCR Master Mix Kit (Applied Biosystems, Foster City, CA, USA) based on the manufacturer's instructions. Each sample was tested in quintuplicate. The reaction conditions were as follows: 10 min at 95 °C (one cycle), 10 s at 95 °C, and 30 s at 60 °C (40 cycles). Gene-specific PCR products were continuously measured using an ABI PRISM 7900 HT Sequence Detection System (PE Applied Biosystems, Norwalk, CT, USA). All the primers used for real-time PCR analysis have been designated using the Primer Express software, version 1.5 (Applied Biosystems, Foster City, CA, USA) and synthesized by the Invitrogen Life Technologies (Carlsbad, CA, USA). The TSHR sequence is as follows: forward, 5′-ATG AGG TAG CAC TGG AGG TC-3′; reverse, 5′-GGC ATC AGG GTC TAT GTA AG-3′. The NIS sequence is as follows: forward, 5′-TCT CAC CCT TTA CCC CTG TG-3′; reverse, 5′- GCG AGC TCC GCG GCC GCG-3′. Normalization was conducted using the differences between the threshold cycle (delta CT) values and GAPDH expression level (forward, 5′-GAC ATG CCG CCT GGA GAA AC-3′; reverse, 5′-AGC CCA GGA TGC CCT TTA GT-3′) to calculate the ET ^delta CT(1 − 2)^/ER ^delta CT(1 − 2)^ ratio, where ET corresponds to the target gene expression level, delta CT^(1 − 2)^ pertains to the differences between the threshold cycle (delta CT) values of the internal control gene and control sample, and ER represents the internal control gene expression level.

### Serum levels of thyroid hormones

The serum free T3, free T4, and TSH concentrations were measured using rat-specific estradiol enzyme-linked immunosorbent assay plates coated with biotin-conjugated binding protein kit purchased from Calbiotech Inc. (Spring Valley, CA, USA). A cardiac puncture was performed, and the blood was centrifuged at 3000 rpm for 30 min. The plasma was separated from the blood that was collected during exsanguination and then immediately frozen in liquid nitrogen and stored at −80 °C.

### Statistical analyses

The data were expressed as means ± standard error of the means. One-way analysis of variance was performed after normal distribution was proved using the Kolmogorov-Smirnov test. Tukey's or Games-Howell post hoc test was used to interpret the results, depending on the outcomes of Levene's test for the homogeneity of variances. A *p* value < 0.05 was considered to be statistically significant.

## References

[1] Sessler DI (2009). Thermoregulatory defense mechanisms. Crit Care Med.

[2] Van Someren EJ (2007). Thermoregulation and aging. Am J Physiol Regul Integr Comp Physiol.

[3] Degroot DW, Kenney WL (2007). Impaired defense of core temperature in aged humans during mild cold stress. Am J Physiol Regul Integr Comp Physiol.

[4] Wagner JA, Robinson S, Marino RP (1974). Age and temperature regulation of humans in neutral and cold environments. J Appl Physiol.

[5] Silva JE (2006). Thermogenic mechanisms and their hormonal regulation. Physiol Rev.

[6] Solmonson A, Mills EM (2016). Uncoupling Proteins and the Molecular Mechanisms of Thyroid Thermogenesis. Endocrinology.

[7] Sellers EA, You SS (1950). Role of the thyroid in metabolic responses to a cold environment. Am J Physiol.

[8] Cannon B, Nedergaard J (2010). Thyroid hormones: igniting brown fat via the brain. Nat Med.

[9] Tabatabaie V, Surks MI (2013). The aging thyroid. Curr Opin Endocrinol Diabetes Obes.

[10] Chahal HS, Drake WM (2007). The endocrine system and ageing. J Pathol.

[11] Kalisnik M (1981). Stereological analysis of the thyroid gland by light microscopy. Prog Clin Biol Res.

[12] Kalisnik M (1972). A histometric thyroid gland activation index (preliminary report). J Microsc.

[13] Rajkovic V, Matavulj M, Gledic D, Lazetic B (2003). Evaluation of rat thyroid gland morphophysiological status after three months exposure to 50 Hz electromagnetic field. Tissue Cell.

[14] Bremner AP, Feddema P, Leedman PJ, Brown SJ, Beilby JP, Lim EM, Wilson SG, O’Leary PC, Walsh JP (2012). Age-related changes in thyroid function: a longitudinal study of a community-based cohort. J Clin Endocrinol Metab.

[15] Kahapola-Arachchige KM, Hadlow N, Wardrop R, Lim EM, Walsh JP (2012). Age-specific TSH reference ranges have minimal impact on the diagnosis of thyroid dysfunction. Clin Endocrinol (Oxf).

[16] Surks MI, Boucai L (2010). Age- and race-based serum thyrotropin reference limits. J Clin Endocrinol Metab.

[17] Danforth E, Burger A (1984). The role of thyroid hormones in the control of energy expenditure. Clin Endocrinol Metab.

[18] Wagner JA, Horvath SM (1985). Influences of age and gender on human thermoregulatory responses to cold exposures. J Appl Physiol.

[19] Westbrook R, Bonkowski MS, Strader AD, Bartke A (2009). Alterations in oxygen consumption, respiratory quotient, and heat production in long-lived GHRKO and Ames dwarf mice, and short-lived bGH transgenic mice. J Gerontol A Biol Sci Med Sci.

[20] Hauck SJ, Hunter WS, Danilovich N, Kopchick JJ, Bartke A (2001). Reduced levels of thyroid hormones, insulin, and glucose, and lower body core temperature in the growth hormone receptor/binding protein knockout mouse. Exp Biol Med (Maywood).

[21] Gesing A, Bartke A, Masternak MM, Lewinski A, Karbownik-Lewinska M (2012). Decreased thyroid follicle size in dwarf mice may suggest the role of growth hormone signaling in thyroid growth regulation. Thyroid Res.

[22] Horvath SM, Radcliffe CE, Hutt BK, Spurr GB (1955). Metabolic responses of old people to a cold environment. J Appl Physiol.

[23] Horvath SM, Rochelle RD (1977). Hypothermia in the aged. Environ Health Perspect.

[24] Silva JE, Bianco SD (2008). Thyroid-adrenergic interactions: physiological and clinical implications. Thyroid.

[25] Silva JE (1995). Thyroid hormone control of thermogenesis and energy balance. Thyroid.

[26] Silva JE (2001). The multiple contributions of thyroid hormone to heat production. J Clin Invest.

[27] Nicoll JB, Gwinn BL, Iwig JS, Garcia PP, Bunn CF, Allison LA (2003). Compartment-specific phosphorylation of rat thyroid hormone receptor alpha1 regulates nuclear localization and retention. Mol Cell Endocrinol.

[28] Tesfamariam B, Waldron T, Seymour AA (1998). Quantitation of tremor in response to beta-adrenergic receptor stimulation in primates: relationship with hypokalemia. J Pharmacol Toxicol Methods.

[29] Sundler F, Grunditz T, Hakanson R, Uddman R (1989). Innervation of the thyroid. A study of the rat using retrograde tracing and immunocytochemistry. Acta Histochem Suppl.

[30] Young JB, Burgi-Saville ME, Burgi U, Landsberg L (2005). Sympathetic nervous system activity in rat thyroid: potential role in goitrogenesis. Am J Physiol Endocrinol Metab.

[31] Ahren B, Bengtsson HI, Hedner P (1986). Effects of norepinephrine on basal and thyrotropin-stimulated thyroid hormone secretion in the mouse. Endocrinology.

[32] Spratt DI, Pont A, Miller MB, McDougall IR, Bayer MF, McLaughlin WT (1982). Hyperthyroxinemia in patients with acute psychiatric disorders. Am J Med.

